# CRISPR/Cas12a-based on-site diagnostics of *Cryptosporidium parvum* IId-subtype-family from human and cattle fecal samples

**DOI:** 10.1186/s13071-021-04709-2

**Published:** 2021-04-20

**Authors:** Fuchang Yu, Kaihui Zhang, Yilin Wang, Dongfang Li, Zhaohui Cui, Jianying Huang, Sumei Zhang, Xiaoying Li, Longxian Zhang

**Affiliations:** 1 College of Animal Science and Veterinary Medicine, Longzihu Campus of Henan Agricultural University, No. 15 Longzihu University Area, Zhengzhou New District, Zhengzhou, 450046 People’s Republic of China; 2 International Joint Research Center for Animal Immunology of China, Zhengzhou, Henan People’s Republic of China

**Keywords:** *Cryptosporidium parvum*, Recombinase polymerase amplification, CRISPR/Cas12a, Visualized detection, On-site detection

## Abstract

**Background:**

*Cryptosporidium parvum* is an enteric protozoan parasite with zoonotic importance and can cause cryptosporidiosis in humans as well as domestic and wild animals worldwide. The IId subtype family (SF) is one of the most prevalent subtypes of *C. parvum*. Some clustered regularly interspaced short palindromic repeats (CRISPR) and CRISPR-associated (Cas) protein systems have been developed to detect nucleic acid with high flexibility, sensitivity and specificity.

**Methods:**

By integrating recombinase polymerase amplification and the Cas12a/crRNA *trans*-cleavage system (termed ReCTC), we established end-point diagnostics by observing fluorescence readouts with the naked eye under blue light and on-site diagnostics using a lateral flow strip (LFS) biosensor.

**Results:**

Our ReCTC-based diagnoses can detect as little as a single copy of a cloned *C. parvum* 60-kDa glycoprotein (GP60) gene, 10 oocysts per gram (OPG), clinical fecal sample without tedious extraction of genomic DNA and have no cross-reactivity with other SFs of *C. parvum* or other common enteric parasitic protozoa.

**Conclusions:**

This study provided a new strategy for direct identification of the IId SF of *C. parvum* free of highly trained operators and expensive special equipment.

**Graphical Abstract:**

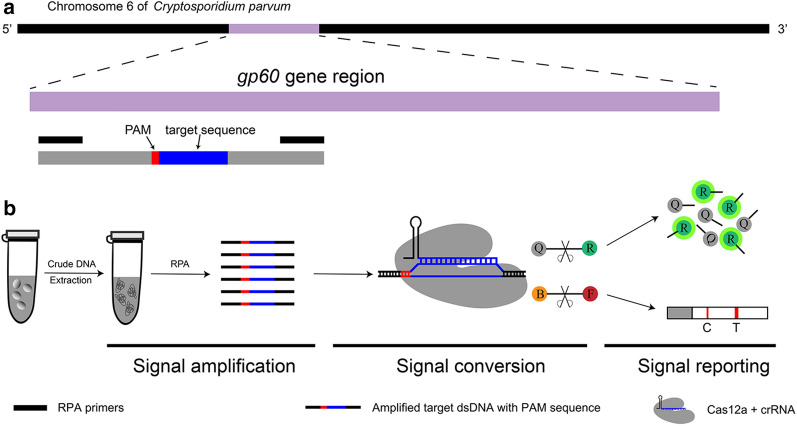

**Supplementary Information:**

The online version contains supplementary material available at 10.1186/s13071-021-04709-2.

## Background

*Cryptosporidium* is an enteric protozoan parasite that can infect various vertebrate species, including humans, nonhuman primates and domestic and wild animals worldwide [1]. It has been demonstrated that cryptosporidiosis is the second greatest cause of diarrhea and mortality in children after rotavirus [2–4]. Currently, 44 valid *Cryptosporidium* species have been identified [5, 6], among which *Cryptosporidium parvum* and *Cryptosporidium hominis* are the predominant species that mainly infect humans and cause > 90% of the human infections [5, 7]. *C. parvum* is also one of the most common species detected in ruminants, especially in young-aged animals [8].

Based on the analysis of the DNA sequence encoding the 60-kDa glycoprotein (GP60), a subtyping tool has been established and most commonly used in studies of the transmission of *C. parvum* in humans and ruminants [7]. A total of 19 subtype families (SFs) (IIa-IIi and IIk-IIt) have been identified for *C. parvum* [5], among which the IId SF is found in both humans and ruminants and is responsible for zoonotic cryptosporidiosis reported in Europe, Middle East, Asia and Australia [7, 9]. Moreover, in China, all *C. parvum* isolates characterized thus far belong to IId subtypes [9].

Because of the common existence and high virulence of the *C. parvum* IId SF, a point-of-care diagnostic method, which can directly distinguish infections caused by the IId SF, is necessary. The diagnosis of *Cryptosporidium* spp. can be divided into the following categories: optical microscopy, antigens or genetic material detection, and the detection of antibodies against *Cryptosporidium* spp. [5]. Among these diagnoses, direct identification of IId SF can only be approached by DNA detection such as PCR amplification combined with nucleotide sequencing [10]. However, this method is time-consuming and complicated, requires highly trained operators and expensive special equipment and is not applicable for on-site clinical disease monitoring, especially in resource-limited areas.

The clustered regularly interspaced short palindromic repeats (CRISPR) and CRISPR-associated (Cas) protein systems have been reported to recognize and cleave specific nucleic acid sequences (namely *cis*-cleavage) [11, 12]. Some Cas proteins, including Cas12a, Cas12b, Cas13a and Cas14, have exhibited nonspecific *trans*-cleavage activity by cleaving non-target sequences when they are activated by recognizing a specific target sequence [13–16]. This collateral effect has been developed into Cas12a/Cas13a-based nucleic acid detection methods termed HOLMES (one-hour low-cost multipurpose highly efficient system), DETECTR (DNA endonuclease-targeted CRISPR trans reporter) and SHERLOCK (specific high-sensitivity enzymatic reporter unlocking) [14, 17, 18]. In DETECTR, CRISPR RNA (crRNA) is designed specifically to target double-strand DNA (dsDNA) located downstream of a short T-rich protospacer-adjacent motif (PAM); therefore, the target DNA works as an activator to trigger both *cis*- and *trans*-cleavage of Cas12a. The fluorophore quencher labeled reporter (FQ Reporter) in the system is then cut, and a fluorescence signal is released and measured [14].

This universal molecular diagnostic methodology has been applied in recent studies to detect African swine fever virus (ASFV) [19, 20] and beta-coronavirus severe acute respiratory syndrome (SARS)-CoV-2 [21], mycoplasma [22] and to identify *Mycobacterium tuberculosis* infection and nontuberculous mycobacteria infection [23]. In the present study, by designing a SF-specific crRNA probe, integrating recombinase polymerase amplification and the Cas12a/crRNA *t*rans-cleavage system (termed ReCTC), we established an end-point diagnostic method by observing fluorescence readouts with the naked eye under blue light and an on-site diagnostic method using a lateral flow strip (LFS) biosensor to detect *C. parvum* IId SF nucleic acid rapidly and accurately.

## Materials and methods

### Reagents and oligonucleotides

All the primers, quenched fluorescent DNA reporter (FAM-TTATT-BHQ1) and lateral flow strip test reporter (FAM-TTATT-biotin) were ordered from Sangon Biotech (Shanghai, China). Partial sequences of *C. parvum gp60* gene of six SFs (IIa–IIf) were synthesized and cloned into pUC57 vectors and transformed into *E. coli* DH5α, respectively (also from Sangon Biotech). All the nucleotide sequences are listed in Additional file 1: Table S1. Recombinant *Francisella novicida* Cas12a (FnCas12a) protein was purchased from Tolo Biotech (Shanghai, China). HiScribe™ T7 High Yield RNA Synthesis Kits were purchased from New England Biolabs (Ipswich, MA, USA). The NucAway™ Spin Columns was purchased from Thermo Fisher Scientific Inc. (Waltham, MA, USA). The TwistAmp® Basic kit was purchased from TwistDx Ltd. (Hertfordshire, UK). The TIANprep mini plasmid kit was purchased from Tiangen Biotech (Shanghai, China). The lateral flow strip biosensor was purchased from Zoonbio Biotechnology (Nanjing, China). Recombinant DNase I (RNase free) and RNase inhibitor were purchased from TaKaRa Bio Inc. (Dalian, China). Other regents used in this study were purchased from Solarbio Science & Technology Co., Ltd. (Beijing, China).

### Cryptosporidium parvum oocyst counting and DNA extraction

Subtype IIdA19G1 oocysts of *C. parvum* were used in this study. Oocysts were purified and counted according to a previous study [24] and stored in phosphate buffer solution (PBS) at 4 °C. N-lauroylsarcosine sodium salt (LSS) was used in the crude DNA extraction, and a homemade DNA banding column was used to purify DNA from the crude extracts (Additional file 1: Figure S1). One μicroliter 10% LSS solution was added to 100 μl PBS containing *C. parvum* oocysts. After intensive mixing, the oocyst suspension was incubated in boiling water for 5 min. Then, heating of the water was stopped and the incubation was retained with the afterheat of boiled water for another 10 min. The lysate was then added to the homemade DNA banding column using a 1-ml syringe and was pushed through the column under the syringe pressure; 100 μl 10 mM Tris solution was added to the column using a new syringe. After incubating for 5 min at room temperature, DNA was eluted in Tris solution under the syringe pressure.

### Design and preparation of crRNA

The *gp60* gene has been used in the identification of SFs of *C. parvum*; thus, we chose it as the target sequence for the design of crRNA. To determine the highly conserved regions with SF specificity, sequences of different SFs were downloaded from GenBank and aligned using ClustalX 2.1 (http://www.clustal.org/clustal2/), and a 24-nucleotide (nt) sequence closely following a T nucleotide-rich protospacer adjacent motif (PAM) was chosen as the target sequence and synthesized containing T7 promotor (Additional file 1: Table S1). The two synthesized reverse complementary crDNA oligonucleotides were annealed to form dsDNA templates, and the crRNA was transcribed using a HiScribe™ T7 High Yield RNA Synthesis Kit followed by DNase I digestion and NucAway™ Spin Column purification. The concentration of purified crRNA was measured using NanoDrop One (Thermo Fisher Scientific Inc., Waltham, MA, USA).

### Recombinase polymerase amplification (RPA) assay

Isothermal recombinase polymerase amplification (RPA) was used to promote the concentration of target dsDNA. Ten pairs of RPA primers were designed (Additional file 1: Table S1), and TwistAmp® Basic kit was used to perform the RPA assay according to the instruction manual. Briefly, the 50-μl total reaction volume was made up of 29.5 μl rehydration buffer, 500 nM of each primer, 5 μl extracted DNA, 2.5 μl 280 nM magnesium acetate (MgOAc) and sterile nuclease-free water up to 50 μl. The reaction was performed at 37 °C for 30 min. The MgOAc was added onto the inner surface of the tube lid while all the other reagents were added to the bottom of the tube that was then carefully capped. A short spin to mix the MgOAc into the solution was applied, which triggered the RPA reactions.

### FnCas12a/crRNA trans-cleavage assay

FnCas12a trans-cleavage assays were performed mainly according to previous studies [14, 25] with some modification. In brief, the 20-μl FnCas12a reaction system included 50 μM FnCas12a, 1 μM purified crRNA, 2 μl target DNA (unpurified RPA products), 1.25 μM collateral ssDNA (FAM-TTATT-BHQ1 reporter for the fluorescence assay or FAM-TTATT-biotin for the lateral flow strip assay), 20 U RNase inhibitor and 2 μl 10 × FnCas12a nuclease reaction buffer. The reaction system was incubated at 37 °C for 1 h, and the fluorescence was detected and imaged with a Tanon-5200 Multi Fluorescence Imager (Tanon Science & Technology Co., Ltd., Tianjin, China). A parallel test was also performed, and the real-time fluorescence value was recorded using a qTOWER^3^G qPCR system (Analytik Jena, Germany) every 5 min.

### Construction of the LFS assay

To enable the on-site diagnosis of *C. parvum* IId SF, a lateral flow readout based on the cleavage of the designed FAM-TTATT-biotin ssDNA reporter was used, which allowed reading the results without sophisticated instruments and skilled technicians. The FAM-TTATT-biotin ssDNA reporter can specifically bind to anti-FITC antibody conjugated with Au nanoparticles and form a complex. When the ssDNA in the reporter is not degraded by FnCas12a, this complex can be captured by the biotin ligand fixed at the control line. On the contrary, when the ssDNA is degraded, biotin is released from the complex, and the complex without biotin cannot be captured by the biotin ligand fixed at the control line but by the IgG antibody at the test line.

In the LFS assay, 20 μl of the ReCTC reaction system was incubated at 37 °C for 1 h followed by mixing with 250 μl of ddH_2_O. Then, 80 μl of the mixture was loaded onto the LFS pad and incubated at room temperature for 3 min. The strips were then photographed using a smartphone camera.

### PCR amplification of *Cryptosporidium parvum* gp60 gene

To assess our ReCTC-based detection, a nested PCR amplification based on the *SSU* rRNA loci of *Cryptosporidium* was used, mainly according to the previous study [26]. Primers used are listed in the Additional file 1: Table S1, and PCR was performed in 25-μl reaction mixtures consisting of 2 μl of DNA preparation (or first PCR product), 1 × KOD-Plus buffer, 200 μM dNTPs, 1 mM MgSO_4_, 300 nM of each primer and 1.5 units of KOD-Plus DNA polymerase (Toyobo, Osaka, Japan). The amplification program consisted of an initial denaturation step at 94 °C for 5 min, followed by 35 cycles of 95 °C for 45 s, 55 °C for 45 s and 72 °C for 1 min, with a final extension of 72 °C for 10 min. Then, the PCR products were analyzed electrophoretically on 1% agarose gel stained with DNAGREEN (Tiandz, Beijing, China) and visualized under UV light. To determine the species and subtype of the *Cryptosporidium* detected, positive secondary PCR products were sequenced bidirectionally by SinoGenoMax Biotechnology Co., Ltd. (Beijing, China), and the generated sequences were aligned in Clustal X software, version 2.1 (http://www.clust al.org/), with reference sequences downloaded from GenBank.

## Results

### Design and preparation of crRNA

**Fig. 1:**
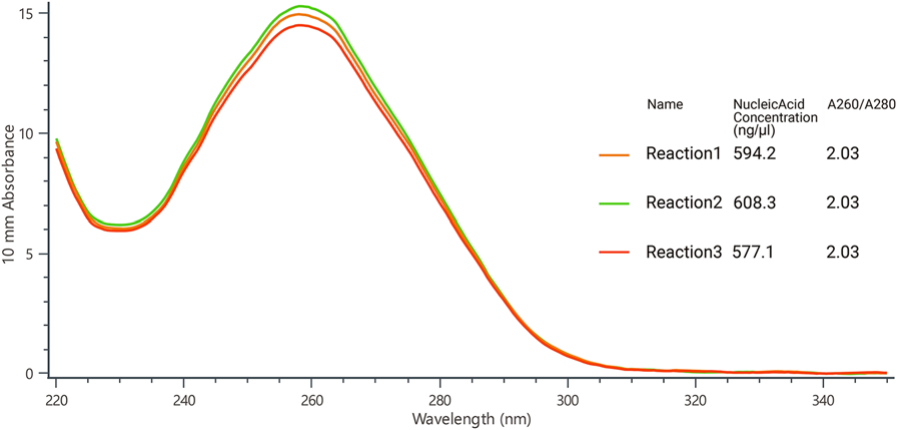
Absorbance curves of purified crRNA. The crRNA was transcribed from crDNA annealed from two reverse complementary single-strand oligonucleotides. The transcribed crRNA dealt with DNase I and was purified using the NucAway™ Spin Column

By multiple alignment of the *gp60* gene, we identified a fragment that was conserved in IId SF but not in other SFs. A 24-nt sequence of this fragment was chosen as the target sequence, and the crRNA sequence was determined (Additional file 1: Table S1; Figure S2). The synthesized single-strand crDNA oligonucleotides were annealed and transcribed to crRNA. Following DNase I digestion and NucAway™ Spin Column purification, the concentration was measured using NanoDrop One. To ensure the stability of the *in vitro* transcription assay, three sets of replications were carried out, and concentrations of the final crRNA ranged from 577.1 to 608.3 ng/μl with a mean concentration of 593.2 ng/μl (Fig. 1 and Additional file 1: Figure S3).

### Optimization of the RPA assay

A number of conserved RPA primers were designed based on the *gp60* gene of *C. parvum* IId SF (GenBank accession no. FJ839877). A set of primers named F01333 and R32535 (Additional file 1: Table S1) showed the highest amplification efficiency by using the TwistAmp Basic Kit (TwistDx, UK) (Additional file 1: Figure S4) and was chosen as the best primer pair. According to the manufacturer's instructions, the optimal reaction temperature was 39 °C. To enable our whole ReCTC assay workflow worked well under 37 °C, we also compared the RPA efficiency at 37 and 39 °C, and the result showed that the primers F01333 and R32535 could work well at both temperatures (Additional file 1: Figure S5).

### Feasibility verification of ReCTC-based detection

**Fig. 2:**
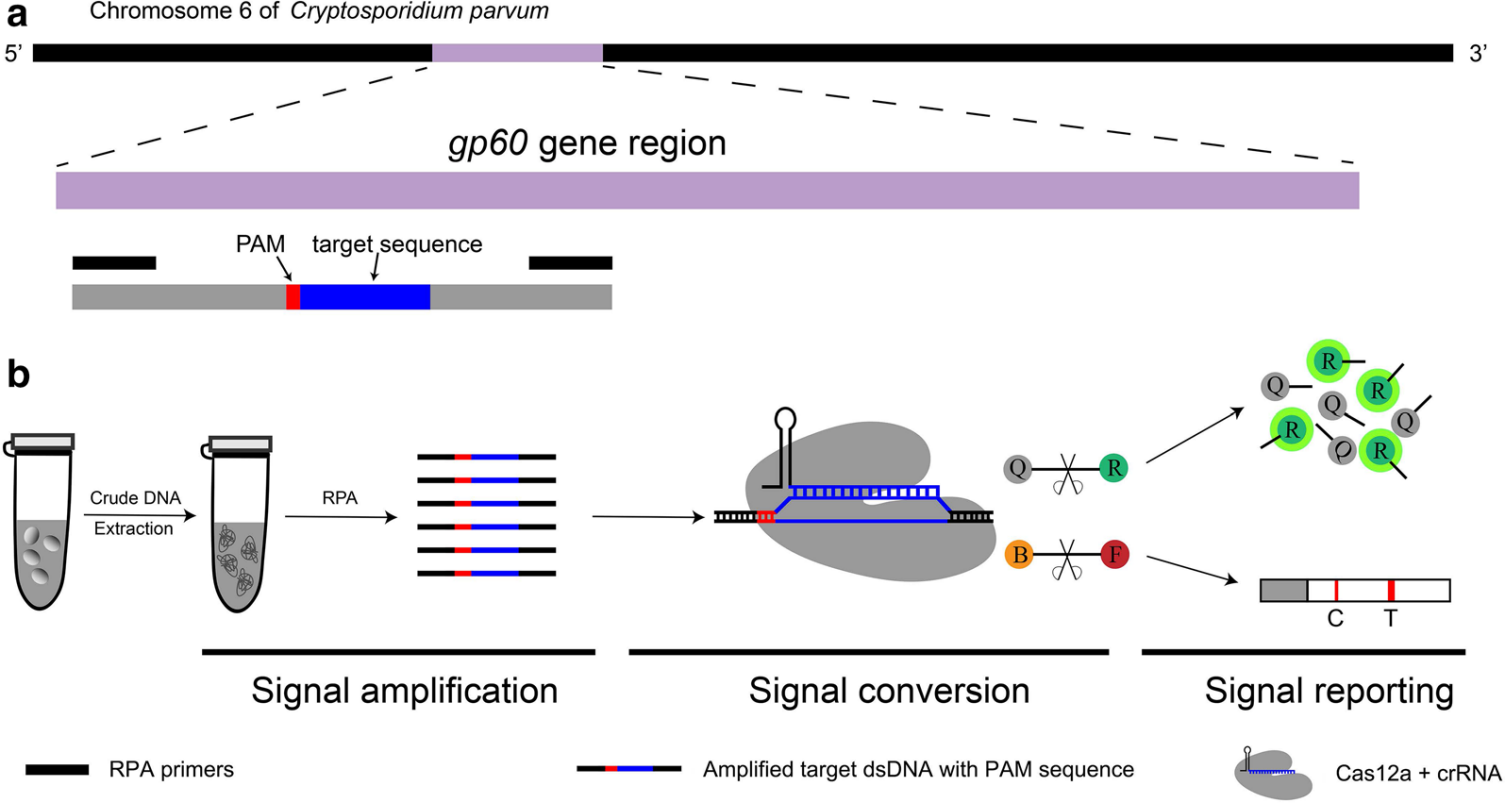
Schematic of the RPA and CRISPR-Cas12a-based detection assay. **a** Diagram of *Cryptosporidium parvum* chromosome 6 showing primers, target sequence and crRNA. RPA primers are indicated by black rectangles; the PAM and target sequences are represented by red and blue rectangles, respectively. **b** Schematic of ReCTC-based diagnosis workflow. The RPA amplicon is used directly as the input of the ReCTC-based detection, and a ternary complex forms if the target DNA exists. *F* fluorophore, *Q* quencher, *B* biotin, *F* FAM

Based on the ReCTC system, we intended to establish the *C. parvum* IId SF detection methods using both the fluorescence and LFS as read-out reporters. Together with the FnCas12a, the designed crRNA and the target dsDNA sequence with a TTN PAM sequence form a triplex. The recognition of the target dsDNA by the designed crRNA can induce the activation of the FnCas12a, which subsequently cleaves the FAM-TTATT-BHQ1 to emit 520-nm fluorescence under 488-nm light or cleaves the FAM-TTATT-biotin reporter to show visible test lines on the LFS (Fig. 2).

**Fig. 3:**
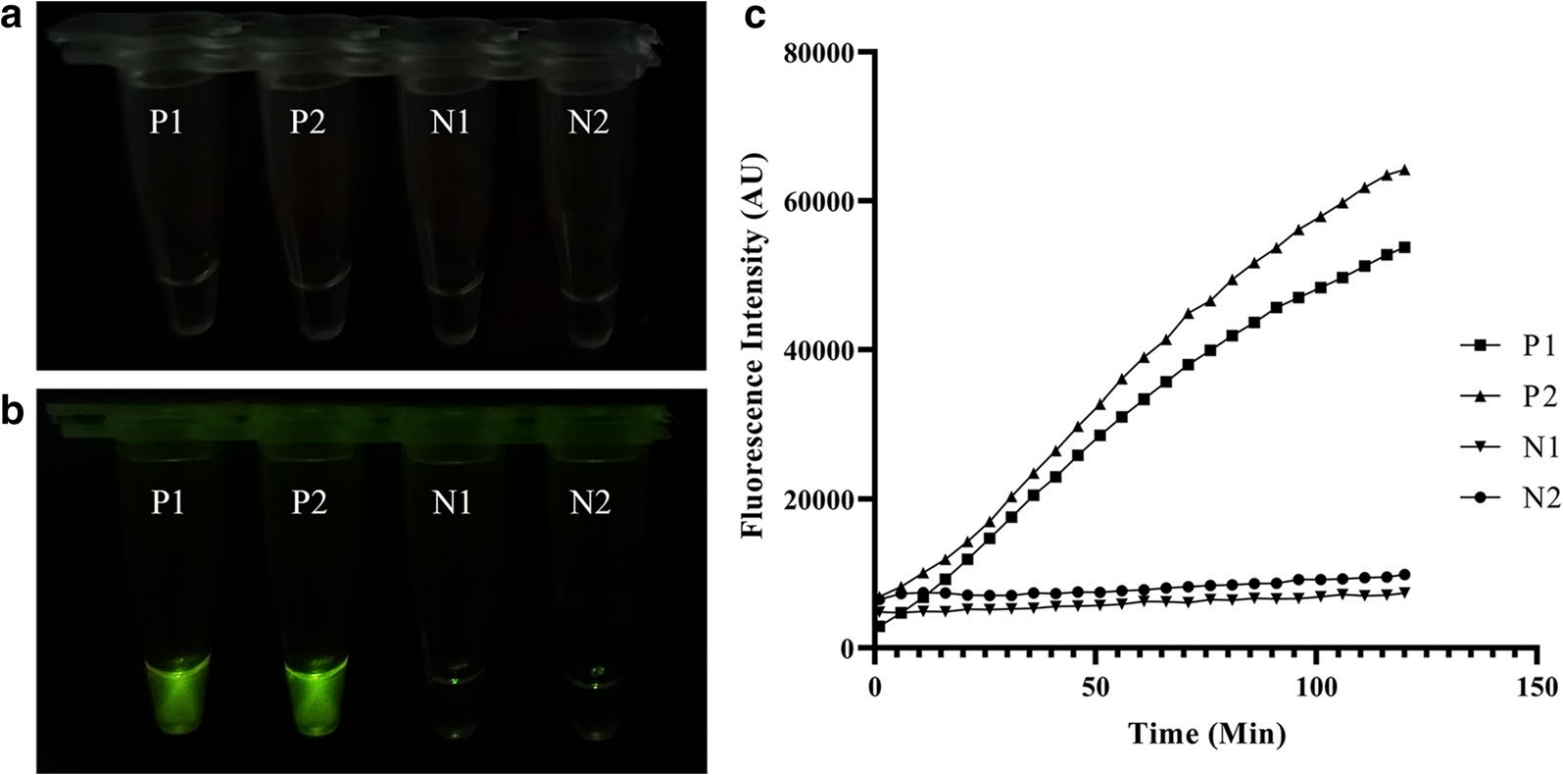
Feasibility verification of the ReCTC-based detection. **a** ReCTC-based fluorescence reaction products showed no signal under visible light. **b** Obvious fluorescence signal can be observed under UV light by the naked eye. P1 and P2: positive results, N1 and N2: negative results. **c** Real-time fluorescence intensity curves of the ReCTC-based detection involving FAM-TTATT-BHQ1 reporter

The fluorescence signal was strong enough to be observed by the naked eye, and distinct differences between positive and negative samples were observed using the CRISPR/Cas12a-based fluorescence detection assay (Fig. 3a, b). To monitor the real-time progress of the reactions and to determine the minimum reaction time in which significant results can be observed, the fluorescence intensity was recorded every 5 min, and the real-time fluorescence curve is shown in Fig. 3c, indicating that 60 min is enough for the observation of the significant difference in fluorescence intensity between positive and negative tests.

**Fig. 4:**
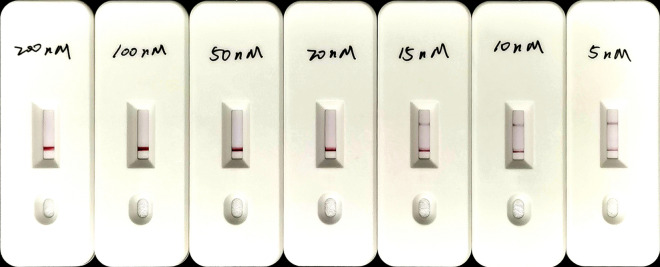
Optimization of the reporter concentration for the ReCTC-based LFS detection. Various concentrations (200, 100, 50, 20, 15, 10, 5 nM) of FAM-TTATT-biotin ssDNA reporter were tested to avoid false-positive and -negative results. The concentrations used were labeled on the LFS pads, and false-positive results wwew eliminated with 20 nM FAM-TTATT-biotin ssDNA reporter or higher concentrations

In the LFS assay, to avoid false-positive results, various concentrations (5–200 nM) of the FAM-TTATT-biotin ssDNA reporter were tested on LFS pads without the addition of any target DNA samples. False-positive results were observed when 15 nM or lower concentrations of the FAM-TTATT-biotin ssDNA reporter were used, and the false-positive effects were eliminated when using concentrations of 20-nM FAM-TTATT-biotin ssDNA reporter or higher (Fig. 4). At 15 nM or lower concentrations, there was not enough FAM-TTATT-biotin ssDNA reporter to combine all the Au nanoparticles labeled by the anti-FITC antibody, so a portion of the nanoparticles that did not bind to the FAM-TTATT-biotin ssDNA reporter kept moving forward until they reached the test band to cause the false-positive result. Therefore, 20 nM was chosen as the optimal concentration of reporter for the ReCTC-based LFS detection.

### Sensitivity of the ReCTC-based detection

**Fig. 5:**
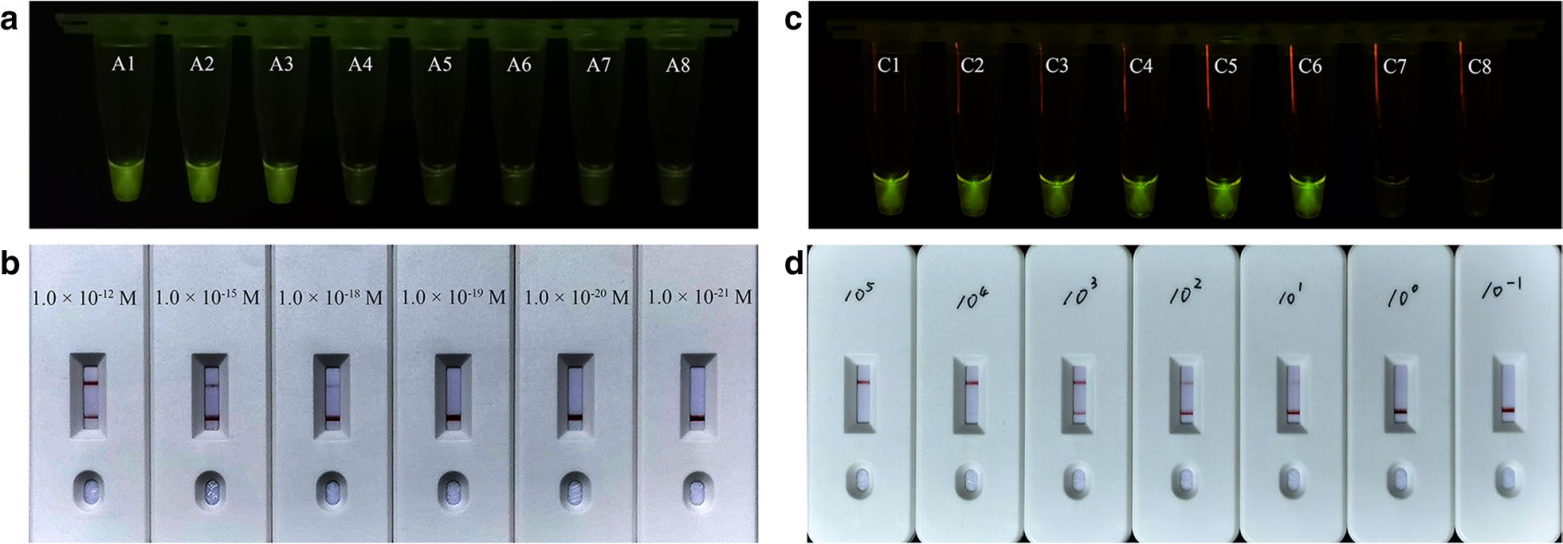
Sensitivity of the ReCTC-based detection. Sensitivity test of ReCTC-based fluorescence (**a**) and LFS (**b**) assay using cloned recombinant plasmid DNA. The LOD of both the fluorescence and LFS assay was determined as 1.0 × 10^–18^ M cloned recombinant plasmid DNA. A1–A8: The concentrations of cloned recombinant plasmid DNA were 1.0 × 10^–12^, 1.0 × 10^–15^, 1.0 × 10^–18^, 1.0 × 10^–19^, 1.0 × 10^–20^, 1.0 × 10^–21^, 1.0 × 10^–22^, 1.0 × 10^–23^ M, respectively. Sensitivity test of ReCTC-based fluorescence (**c**) and LFS (**d**) assay using crude DNA extracted from purified oocysts. The LOD of both the fluorescence and LFS assay was determined as one and ten oocysts per milliliter, respectively. C1–C8: The numbers of oocysts per milliliter were equivalent to 1 × 10^5^, 1 × 10^4^, 1 × 10^3^, 1 × 10^2^, 1 × 10^1^, 1, 0.1 and 0, respectively. The concentrations of cloned recombinant plasmid DNA and the numbers of oocysts per milliliter used in the sensitivity test of LFS assay (**b**, **d**) were indicated on the LFS pads

The limit of detection (LOD) of the ReCTC was evaluated using a cloned *C. parvum* IId family *gp60* gene with a conserved PAM (TTC) region cloned in pUC57 plasmid (Additional file 1: Table S2) and verified with PCR amplification. The cloned plasmid DNA was serially diluted to different concentrations and subsequently applied to the ReCTC. Results of fluorescence detection showed that samples with concentrations of 1.0 × 10^–18^ M or higher target plasmid DNA showed an obvious fluorescence signal compared to samples with lower concentrations and negative control (Fig. 5a). Similar results were also observed in the ReCTC-based LFS detection. The lowest concentration of samples that showed a test line was 1.0 × 10^–18^ M, indicating the LOD of the ReCTC-based LFS detection was also 1.0 × 10^–18^ M target plasmid DNA (Fig. 5b).

The sensitivity of the ReCTC-based fluorescence detection and LFS detection was also tested using crude DNA extracted from purified oocysts. The concentration of purified oocysts was adjusted to 1 × 10^6^ oocysts per milliliter before the DNA extraction. The concentration of extracted DNA was 9.3 ng/μl and serially diluted to concentrations of 9.3 × 10^–7^ to 9.3 × 10^–1^ ng/μl, which were equivalent to 0.1 to 1 × 10^5^ oocysts per milliliter, respectively. In the ReCTC-based fluorescence detection, samples with crude genomic DNA of 9.3 × 10^–6^ or higher showed observable fluorescence signals, representing an LOD of one oocyst per milliliter (Fig. 5c and Additional file 1: Figure S6). In the ReCTC-based LFS detection assay, the sample with 9.3 × 10^–5^ ng/μl DNA showed a faint test line and samples with higher input target DNA showed strong test lines, indicating that ten oocysts per milliliter was detected with the ReCTC-based LFS detection (Fig. 5d).

### Specificity of the ReCTC-based detection

**Fig. 6:**
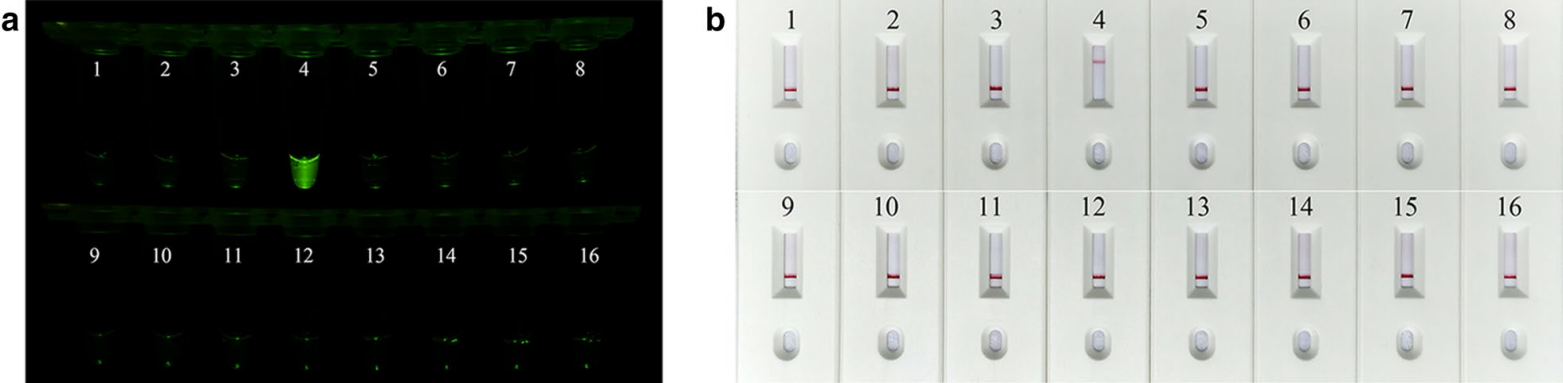
Specificity of the ReCTC-based detection. Recombinant pUC57 plasmid DNA containing *gp60* gene of IIa, IIb, IIc, IId, IIe and IIf SFs of *C. parvum* (1–6) and genomic DNA of *C. andersoni*, *C. hominis*, *C. meleagridis*, *C. muris*, *C. bovis*, *C. ryanae*, *Enterocytozoon bieneusi*, *Giardia duodenalis*, *Blastocystis hominis* and *Cyclospora cayetan* (7–16) were included. **a** Specificity test of the ReCTC-based fluorescence detection assay. Only the sample of *C. parvum* IId SF exhibited a strong fluorescence signal. **b** Specificity test of the ReCTC-based LFS detection assay. A clear test line was observed only on the LFS where *C. parvum* IId SF recombinant pUC57 plasmids DNA was added

The specificity of the ReCTC-based detection was verified using the recombinant pUC57 plasmid DNA containing the *gp60* gene of six different *C. parvum* SFs (IIa, IIb, IIc, IId, IIe and IIf) (Additional file 1: Table S2). Genomic DNA extracted from several prevalent *Cryptosporidium* species (*C. andersoni*, *C. hominis*, *C. meleagridis*, *C. muris*, *C. bovis* and *C. ryanae*) and other intestinal protozoa (*Enterocytozoon bieneusi*, *Giardia duodenalis*, *Blastocystis hominis* and *Cyclospora cayetanensis*) were also included in the specificity test. As shown in Fig. 6a, only samples of *C. parvum* IId SF exhibited strong fluorescence signal, which could be observed by the naked eye and verified by the qTOWER^3^G real-time PCR system (Additional file 1: Figure S7). The LFS detection further confirmed the specificity of the ReCTC, and a clear test line was observed only on the LFS where *C. parvum* IId SF recombinant pUC57 plasmids DNA was added (Fig. 6b).

### Performance of the ReCTC-based detection of *C. parvum* IId SF on clinical samples

**Fig. 7:**
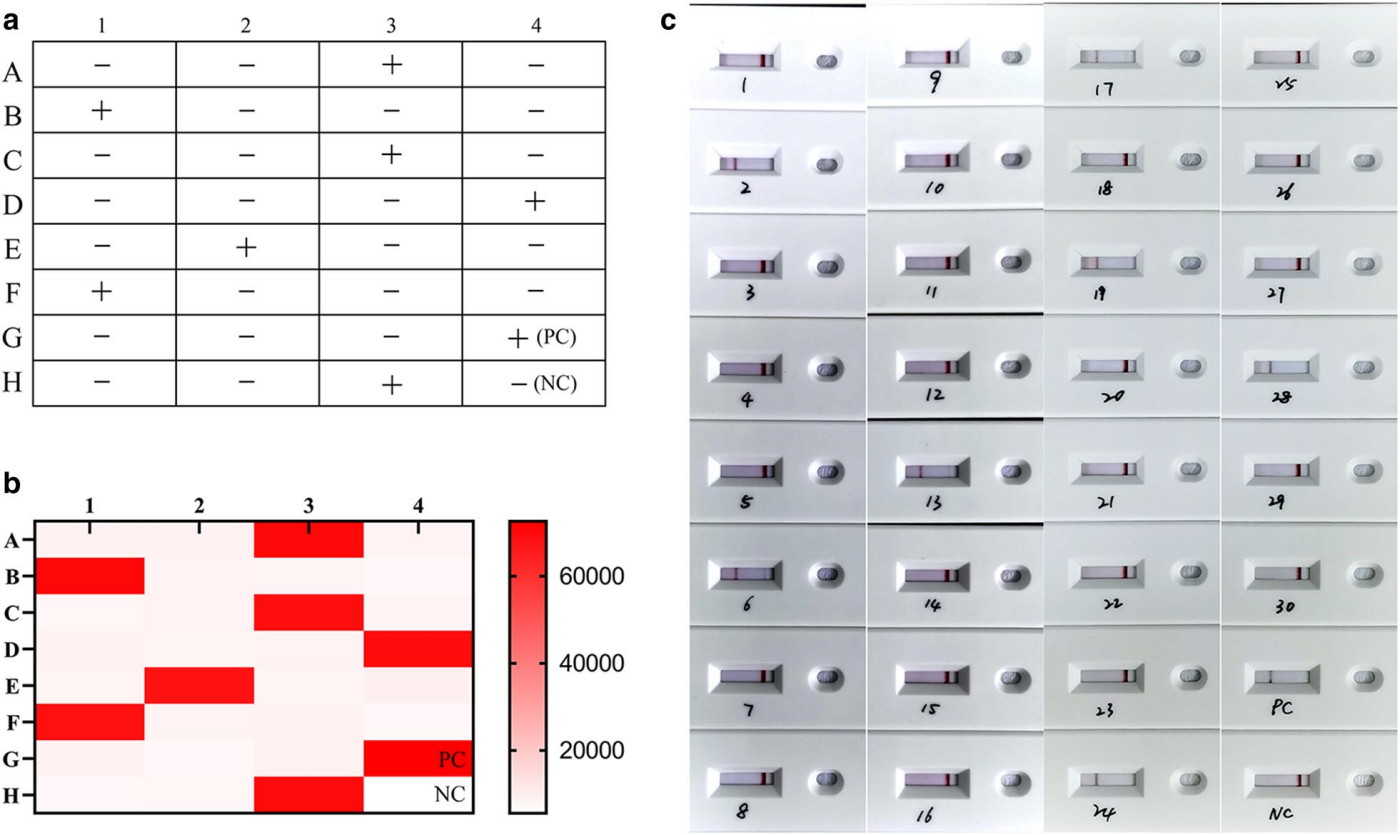
Validation of ReCTC-based detection of *C. parvum* IId SF in clinical cattle samples. Clinical fecal samples from 30 dairy cattle were tested by **a** conventional nested PCR sequencing method, **b** our ReCTC-based fluorescence and **c** LFS detection. Both the ReCTC-based fluorescence and LFS detection agreed 100% with the conventional nested PCR sequencing method

To assess the clinical utility of the ReCTC-based detection, fresh fecal specimens were collected from 30 preweaned calves. The specimens were filtered through a 7.62-cm-diameter sieve with a pore size of 45 μm and applied to the crude DNA extraction. The extracted crude DNA samples were initially screened using nested PCR based on the *gp60* gene of *C. parvum*, and results of sequencing and alignment revealed that the positive rate of *C. parvum* IId SF was 23.3% (7/30) (Fig. 7a). All the crude DNA samples were then tested by ReCTC-based detection. Consistent with the PCR results, distinct fluorescence signals were observed in all the PCR-positive samples by the naked eye and no such signal in all the PCR-negative samples, showing an accordance rate of 100% with PCR-based nucleotide sequencing (Fig. 7b). The ReCTC-based LFS detection also was in 100% accordance with the conventional PCR sequencing method (Fig. 7c).

**Fig. 8:**
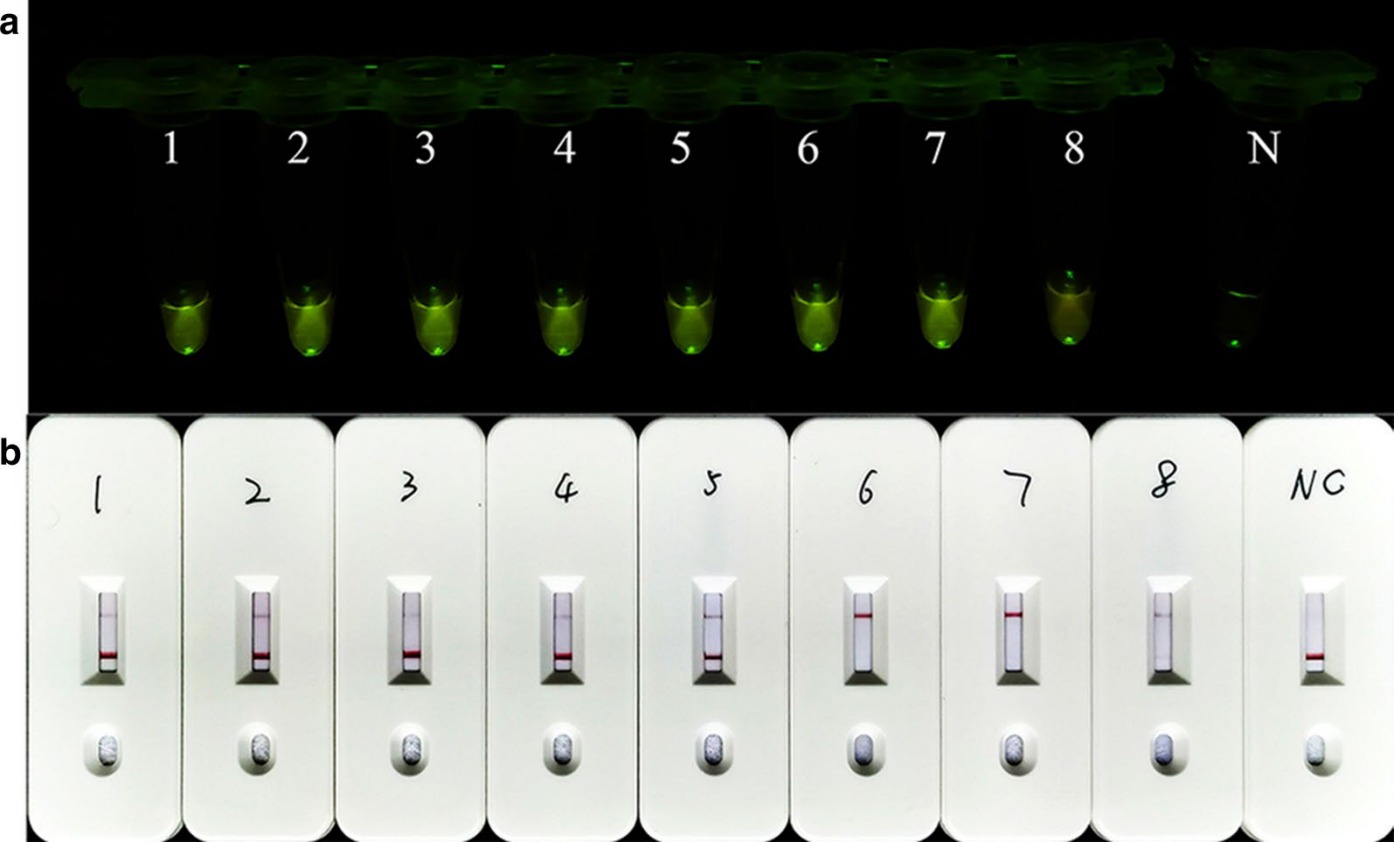
Validation of ReCTC-based detection of *C. parvum* IId SF in positive clinical human samples. Clinical human fecal DNA samples collected from inpatients that had been identified as positive for *C. parvum* IIdA19G1 were subjected to **a** ReCTC-based fluorescence and **b** LFS detection. *N* negative control

To validate the ReCTC diagnostic accuracy in human samples, eight clinical DNA samples from diarrhea patients that had been detected as positive for *C. parvum* IId SF were tested using ReCTC-based detection. Fluorescence signals were observed in all eight samples by the naked eye in the ReCTC-based fluorescence assay (Fig. 8a). The positive results were also confirmed by the fluorescence values recorded by the qTOWER^3^G qPCR system (Additional file 1: Figure S8). In the ReCTC-based LFS assay, all eight clinical DNA samples showed the test bands on the LFS pads (Fig. 8b).

## Discussion

In this study, we combined isothermal recombinase polymerase amplification with the Cas12a/crRNA trans-cleavage system to establish the ReCTC method and used fluorescence or chromatography readout as signal reporting. We found that ReCTC was able to detect *C. parvum* IId SF with high sensitivity and specificity. The use of CRISPR/Cas12a to detect infectious antigens has been reported in many other studies on detecting ASFV [20], human papillomavirus (HPV) [14] and the recently emerged coronavirus, SARS-CoV-2 [21]. Here, we first applied this method in detection of the zoonotic parasitic protozoan *C. parvum*, and the ReCTC-based fluorescence or LFS assay exhibited advantages in point-of-care use without the need for technical expertise, ancillary equipment or power (LFS assay).

In previous Cas12a off-target studies, a mismatch tolerance pattern was observed [27, 28], and PAM mutations or mismatches in the PAM-adjacent region inhibited *trans*-cleavage when the activator was a dsDNA [14]. This stringent principle gives the Cas12a *trans* cleavage-based diagnosis good specificity, which was confirmed in our specificity test with several other common intestinal pathogens and other subtypes of *C. parvum*.

The currently reported CRISPR/Cas12a biosensing systems generally consist of three key parts: signal amplification, signal transducing and signal reporting [29]. In our ReCTC-based diagnosis, the RPA was chosen as the signal amplification method for its mild temperature requirements. Our results indicated that the RPA assay worked at 37 °C as well as 39 °C, which made the ReCTC more convenient as the downstream Cas12a *trans*-cleavage system was also conducted at 37 °C. A water/metal bath kettle, constant temperature incubator or even body heating can carry out the ReCTC reaction.

Two elements were used in the signal reporting part in this study: a fluorescence readout and LFS biosensor, corresponding to different reporters, FAM-TTATT-BHQ1 and FAM-TTATT-biotin, respectively. When the FAM-TTATT-BHQ1 reporter was used, bright green fluorescence signals could be observed by the naked eye under blue light with a Tanon-5200 Multi Fluorescence Imager. In resource-limited areas, a much cheaper device, Blue Light Gel Imager (Sangon Biotech. Inc., Shanghai, China; 440–485 nm), is recommended [22]. When FAM-TTATT-biotin was used in the signal reporting part, the readout could be observed using a LFS biosensor, which did not rely on technical expertise, ancillary equipment or power. This trait makes ReCTC-based LFS detection a real on-site diagnostic tool for *C. parvum* IId SF in field conditions.

As a biosensor, the CRISPR/Cas12a/crRNA system acts as a signal converter, converting the existence of target dsDNA to the presence of fluorescence or colorimetric signals by cutting the collateral ssDNA reporter [29]. Once the high endonuclease activity of FnCas12a was activated by the amplified target dsDNA, it could cleave a large number of collateral ssDNA reporters [25]. Together with RPA, the signal amplification part, the efficient cleavage activity of FnCas12a makes ReCTC-based detection highly sensitive.

In conclusion, by integrating recombinase polymerase amplification and Cas12a/crRNA *t*rans-cleavage (termed ReCTC), we established an end point diagnostic method by observing fluorescence readouts with the naked eye under blue light and an on-site diagnostic method using a lateral flow strip (LFS) biosensor. Our ReCTC-based diagnoses could detect the IId subtype family of *C. parvum* from clinical fecal samples independent of professional technicians, expensive instruments or cumbersome operations. The newly established ReCTC-based detection displayed one and ten copy sensitivity in pure and complex samples, respectively, and the specificity was also confirmed to be robust. The ReCTC-based detection also agreed 100% with the conventional PCR sequencing method, which is the current gold standard commonly used in the diagnosis of *C. parvum* IId SF. Further optimization of the ReCTC assay as a one-pot reaction should be done in our future research to detect *C. parvum* IId SF from the clinical samples in the field more rapidly and simply.

## Supplementary Information

**Additional file 1**: **Figure S1**. Absorbance curves of purified crRNA. The crRNA was transcribed from crDNA annealed from two reverse complementary single-strand oligonucleotides. The transcribed crRNA was treated with DNase I and was purified using the NucAway™ Spin Column. **Figure S2**. Schematic of the RPA and CRISPR-Cas12a-based detection assay. A. Diagram of *Cryptosporidium parvum* chromosome 6 showing primers, target sequence and crRNA. RPA primers are indicated by black rectangles; the PAM and target sequences are represented by red and blue rectangles, respectively. B. Schematic of ReCTC-based diagnostic workflow. The RPA amplicon is used directly as the input of the ReCTC-based detection, and a ternary complex forms if the target DNA exists. F, fluorophore; Q, quencher; B, biotin; F, FAM. **Figure S3**. Feasibility verification of the ReCTC-based detection. A. ReCTC-based fluorescence reaction products showed no signal under visible light. B. Obvious fluorescence signal can be observed under UV light by the naked eye. P1 and P2: positive results; N1 and N2: negative results. C. The real-time fluorescence intensity curves of the ReCTC-based detection involving FAM-TTATT-BHQ1 reporter. **Figure S4**. Optimization of reporter concentration for the ReCTC-based LFS detection. Various concentrations (200, 100, 50, 20, 15, 10, 5 nM) of FAM-TTATT-biotin ssDNA reporter were tested to avoid false-positive and -negative results. The concentrations used were labeled on the LFS pads, and false-positive results were eliminated with 20 nM or higer FAM-TTATT-biotin ssDNA reporter concentrations. **Figure S5**. Sensitivity of the ReCTC-based detection. A, B. Sensitivity test of ReCTC-based fluorescence (A) and LFS (B) assay using cloned recombinant plasmid DNA. The LOD of both the fluorescence and LFS assay was determined as 1.0 × 10-18 M cloned recombinant plasmid DNA. A1–A8: The concentrations of cloned recombinant plasmid DNA were 1.0 × 10-12, 1.0 × 10-15, 1.0 × 10-18, 1.0 × 10-19, 1.0 × 10-20, 1.0 × 10-21, 1.0 × 10-22, 1.0 × 10-23 M, respectively. C, D. Sensitivity test of ReCTC-based fluorescence (C) and LFS (D) assay using crude DNA extracted from purified oocysts. The LOD of both the fluorescence and LFS assay was determined as one and ten oocysts per milliliter, respectively. C1–C8: The numbers of oocysts per milliliter were equivalent to 1 × 105, 1 × 104, 1 × 103, 1 × 102, 1 × 101, 1, 0.1 and 0, respectively. The concentrations of cloned recombinant plasmid DNA and the numbers of oocysts per milliliter used in the sensitivity test of the LFS assay (B, D) were indicated on the LFS pads. **Figure S6**. Specificity of the ReCTC-based detection. Recombinant pUC57 plasmid DNA containing the gp60 gene of IIa, IIb, IIc, IId, IIe and IIf SFs of *C. parvum* (1–6) and genomic DNA of *C. andersoni, C. hominis, C. meleagridis, C. muris, C. bovis, C. ryanae, Enterocytozoon bieneusi, Giardia duodenalis, Blastocystis hominis* and *Cyclospora cayetan* (7–16) were included. A. Specificity test of the ReCTC-based fluorescence detection assay. Only the sample of *C. parvum* IId SF exhibited a strong fluorescence signal. B. Specificity test of the ReCTC-based LFS detection assay. A clear test line was observed only on the LFS where *C. parvum* IId SF recombinant pUC57 plasmid DNA was added. **Figure S7**. ReCTC-based detection of *C. parvum* IId SF on clinical cattle samples. Clinical fecal samples from 30 dairy cattle were tested by a conventional nested PCR sequencing method (A) and our ReCTC-based fluorescence (B) and LFS (C) detection. Both the ReCTC-based fluorescence and LFS detection agreed 100% with the conventional nested PCR sequencing method. **Figure S8**. ReCTC-based detection of *C. parvum* IId SF on positive clinical human samples. Clinical human fecal DNA samples collected from inpatients that had been identified as positive for *C. parvum* IIdA19G1 were subjected to ReCTC-based fluorescence (A) and LFS (B) detection. N: negative control. **Table S1**. Nucleotide sequences used in this study. **Table S2**. Partial sequences of *C. parvum* gp60 gene of six subtype families (IIa–IIf) cloned into the pUC57 vectors.

## Data Availability

The data that support the findings of this study are available from the corresponding author, Longxian Zhang: zhanglx8999@henau.edu.cn.

## Abbreviations

CRISPR: Clustered regularly interspaced short palindromic repeats
Cas: CRISPR-associated
SF: Subtype family
ReCTC: Recombinase polymerase amplification and Cas12a/crRNA trans-cleavage
LFS: Lateral flow strip
GP60: 60 KDa glycoprotein
OPG: Oocysts per gram
PCR: Polymerase chain reaction
HOLMES: One-hour low-cost multipurpose highly efficient system
DETECTR: DNA endonuclease-targeted CRISPR trans reporter
SHERLOCK: Specific high-sensitivity enzymatic reporter unlocking
crRNA: CRISPR RNA
dsDNA: Double-strand DNA
PAM: Protospacer-adjacent motif
FQ Reporter: Fluorophore quencher labeled reporter
ASFV: African swine fever virus
SARS-CoV-2: Beta-coronavirus severe acute respiratory syndrome
PBS: Phosphate buffer solution
LSS: N-Lauroylsarcosine sodium salt
RPA: Recombinase polymerase amplification
LOD: Limit of detection
HPV: Human papillomavirus
