# Supplementary material for: CRISPR/Cas12a-based on-site diagnostics of Cryptosporidium parvum IId-subtype-family from human and cattle fecal samples
Source: Parasit Vectors. 2021 Apr 20;14:208. doi: 10.1186/s13071-021-04709-2 (PMC8056104; doi:10.1186/s13071-021-04709-2)
Supplement: Supplementary file 1 — Additional file 1: Figure S1. Absorbance curves of purified crRNA. The crRNA was transcribed from crDNA annealed from two reverse complementary single-strand oligonucleotides. The transcribed crRNA was treated with DNase I and was purified using the NucAway™ Spin Column. Figure S2. Schematic of the RPA and CRISPR-Cas12a-based detection assay. A. Diagram of Cryptosporidium parvum chromosome 6 showing primers, target sequence and crRNA. RPA primers are indicated by black rectangles; the PAM and target sequences are represented by red and blue rectangles, respectively. B. Schematic of ReCTC-based diagnostic workflow. The RPA amplicon is used directly as the input of the ReCTC-based detection, and a ternary complex forms if the target DNA exists. F, fluorophore; Q, quencher; B, biotin; F, FAM. Figure S3. Feasibility verification of the ReCTC-based detection. A. ReCTC-based fluorescence reaction products showed no signal under visible light. B. Obvious fluorescence signal can be observed under UV light by the naked eye. P1 and P2: positive results; N1 and N2: negative results. C. The real-time fluorescence intensity curves of the ReCTC-based detection involving FAM-TTATT-BHQ1 reporter. Figure S4. Optimization of reporter concentration for the ReCTC-based LFS detection. Various concentrations (200, 100, 50, 20, 15, 10, 5 nM) of FAM-TTATT-biotin ssDNA reporter were tested to avoid false-positive and -negative results. The concentrations used were labeled on the LFS pads, and false-positive results were eliminated with 20 nM or higer FAM-TTATT-biotin ssDNA reporter concentrations. Figure S5. Sensitivity of the ReCTC-based detection. A, B. Sensitivity test of ReCTC-based fluorescence (A) and LFS (B) assay using cloned recombinant plasmid DNA. The LOD of both the fluorescence and LFS assay was determined as 1.0 × 10^-18^ M cloned recombinant plasmid DNA. A1–A8: The concentrations of cloned recombinant plasmid DNA were 1.0 × 10^-12^, 1.0 × 10^-15^, 1.0 × 10^-18^, 1.0 × 10^-19^, 1.0 × 10 [file 13071_2021_4709_MOESM1_ESM.docx]

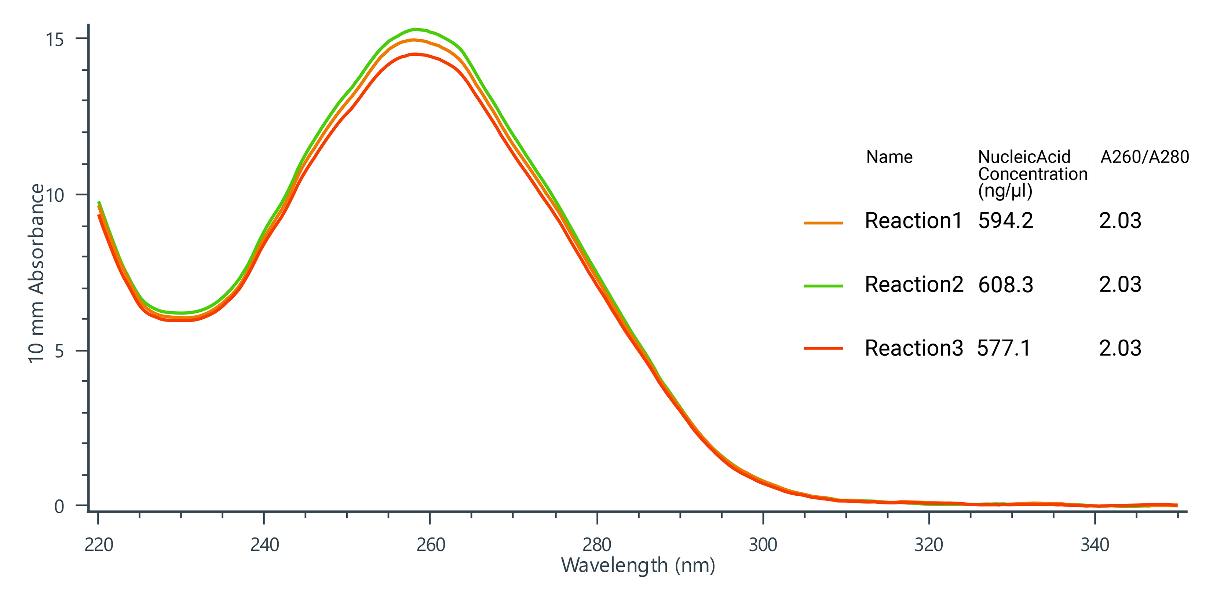


**Figure 1. Absorbance curves of purified crRNA**. The crRNA was transcribed from crDNA annealed from two reverse complementary single strand oligonucleotides. The transcribed crRNA was dealt with DNase I and was purified using the NucAway™ Spin Column.


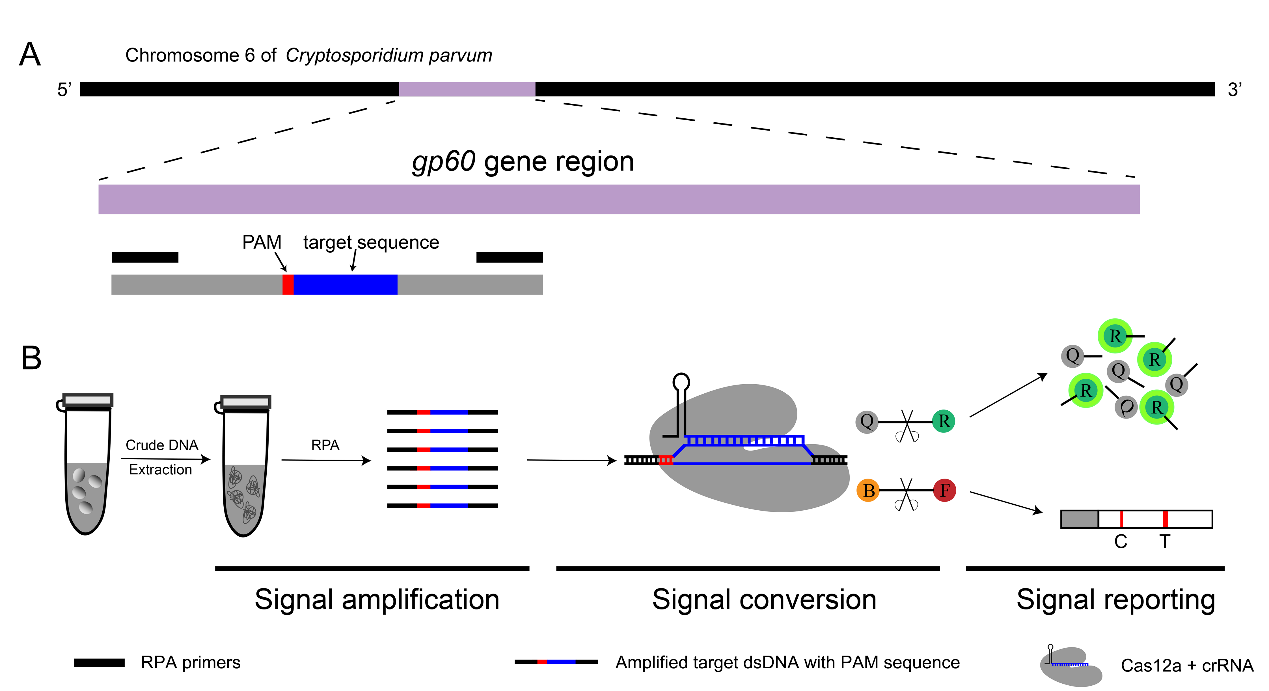


**Figure 2. Schematic of the RPA and CRISPR-Cas12a based detection assay**. **A**. Diagram of *Cryptosporidium parvum* chromosome 6 showing primers, target sequence and crRNA. RPA primers are indicated by black rectangles, the PAM and target sequences are represented by red and blue rectangles, respectively. **B**. Schematic of ReCTC based diagnoses workflow. The RPA amplicon is used directly as the input of the ReCTC based detection, and a ternary complex forms if the target DNA exists. F, fluorophore; Q, quencher; B, Biotin; F, FAM.


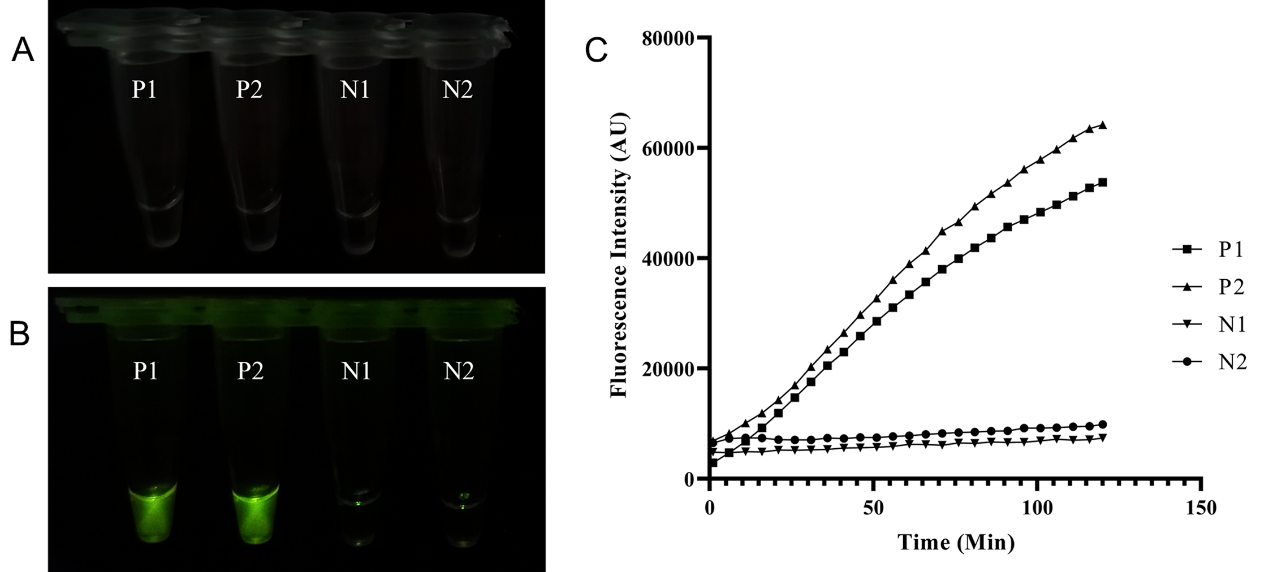


**Figure 3. Feasibility verification of the ReCTC based detection**. **A**. ReCTC based fluorescence reaction products showed no signal under the visible light. **B**. Obvious fluorescence signal can be observed under the UV light by naked eyes. P1 and P2: positive results, N1 and N2: negative results. **C**. The real-time fluorescence intensity curves of the ReCTC based detection involving FAM-TTATT-BHQ1 reporter.


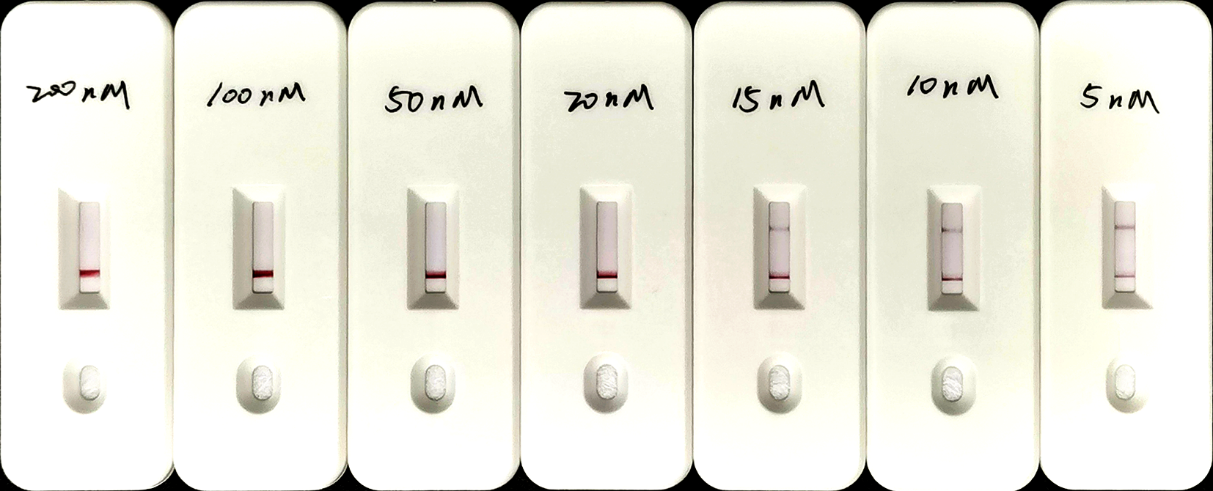


**Figure 4. Optimization of reporter concentration for the ReCTC based LFS detection**. Various concentrations (200, 100, 50, 20, 15, 10, 5 nM) of FAM-TTATT-Biotin ssDNA reporter were tested to avoid false-positive and false-negative result. The concentrations used were labeled on the LFS pads and false-positive result was eliminated with 20 nM FAM-TTATT-Biotin ssDNA reporter or higher concentrations.


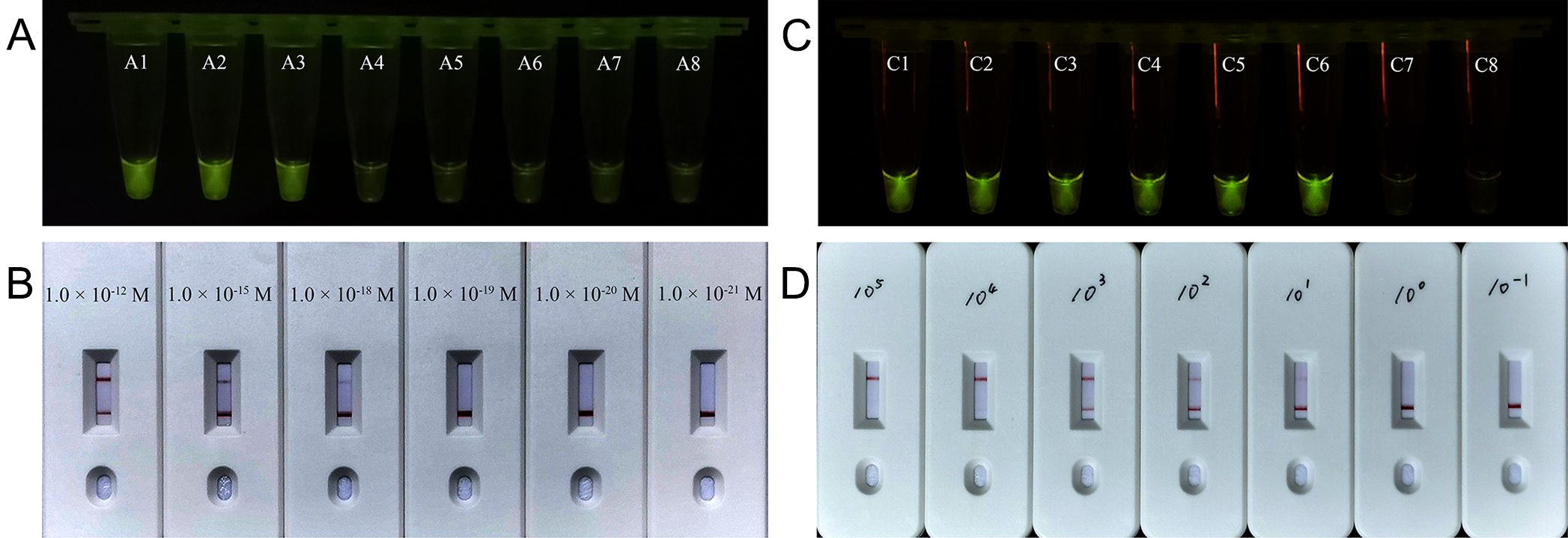


**Figure 5. Sensitivity of the ReCTC based detection**. **A**, **B**. Sensitivity test of ReCTC based fluorescence (**A**) and LFS (**B**) assay using cloned recombinant plasmid DNA. The LOD of both fluorescence and LFS assay were determined as 1.0 × 10^-18^ M cloned recombinant plasmid DNA. A1-A8: The concentrations of cloned recombinant plasmid DNA were 1.0 × 10^-12^, 1.0 × 10^-15^, 1.0 × 10^-18^, 1.0 × 10^-19^, 1.0 × 10^-20^, 1.0 × 10^-21^, 1.0 × 10^-22^, 1.0 × 10^-23^ M, respectively. **C**, **D**. Sensitivity test of ReCTC based fluorescence (**C**) and LFS (**D**) assay using crude DNA extracted from purified oocysts. The LOD of both fluorescence and LFS assay were determined as one and ten oocysts per milliliter, respectively. C1-C8: The numbers of oocysts per milliliter were equivalent to 1 × 10^5^, 1 × 10^4^, 1 × 10^3^, 1 × 10^2^, 1 × 10^1^, 1, 0.1 and 0, respectively. The concentrations of cloned recombinant plasmid DNA and the numbers of oocysts per milliliter used in the sensitivity test of LFS assay (**B**, **D**) were indicated on the LFS pads.


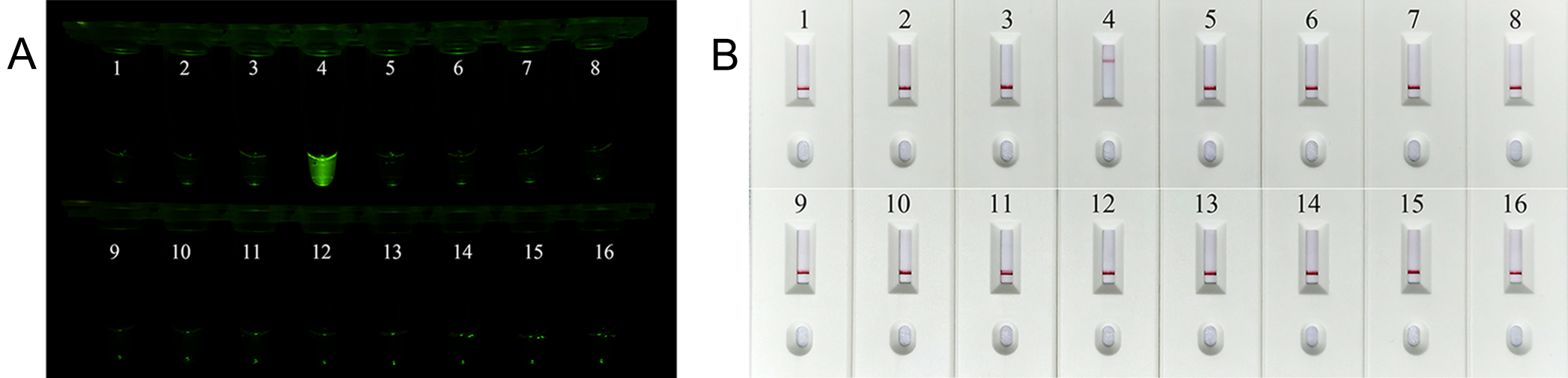


**Figure 6. Specificity of the ReCTC based detection**. Recombinant pUC57 plasmids DNA containing *gp60* gene of IIa, IIb, IIc, IId, IIe and IIf SFs of *C. parvum* (1-6) and genomic DNA of *C. andersoni*, *C. hominis*, *C. meleagridis*, *C. muris*, *C. bovis*, *C. ryanae*, *Enterocytozoon bieneusi*, *Giardia duodenalis*, *Blastocystis hominis* and *Cyclospora cayetan* (7-16) were included. **A**. Specificity test of the ReCTC based fluorescence detection assay. Only sample of *C. parvum* IId SF exhibited strong fluorescence signal. **B**. Specificity test of the ReCTC based LFS detection assay. Clear test line was observed only on the LFS that *C. parvum* IId SF recombinant pUC57 plasmids DNA was added.


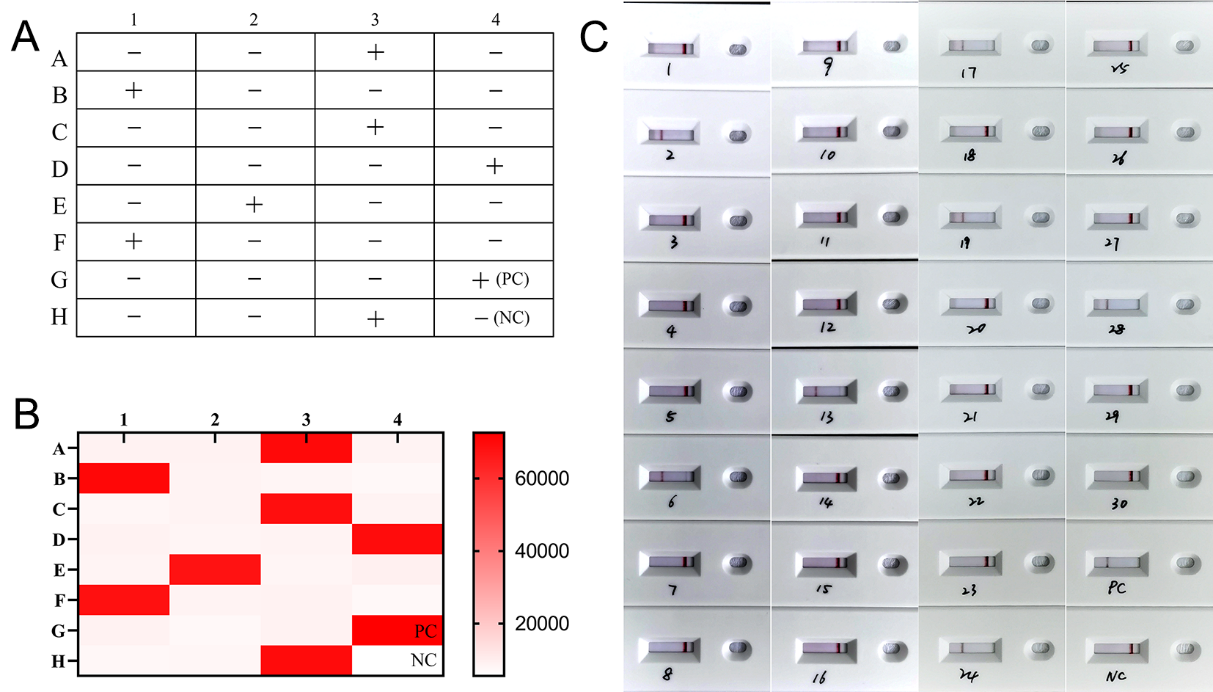


**Figure 7. ReCTC based detection of *C. parvum* IId SF on clinical cattle samples**. Clinical fecal samples from 30 dairy cattle were tested by conventional nested PCR-sequencing method (**A**) and our ReCTC based fluorescence (**B**) and LFS (**C**) detection. Both the ReCTC based fluorescence and LFS detection corroborated 100% with the conventional nested PCR-sequencing method.


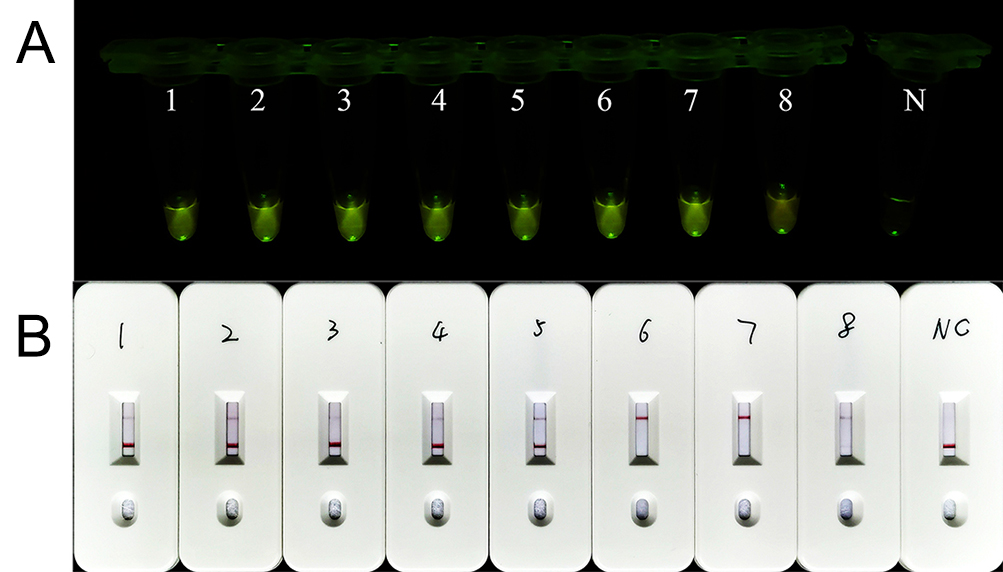


**Figure 8. ReCTC based detection of *C. parvum* IId SF on positive clinical human samples.** Clinical human fecal DNA samples collected from inpatients that had been identified as positive for *C. parvum* IIdA19G1 were subjected to the ReCTC based fluorescence (**A**) and LFS (**B**) detection. N: negative control.

Table S1. Nucleotide sequences used in this study

| Name | | Sequence (5’-3’) |
| --- | --- | --- |
| Target dsDNA (*C. parvum* IIdA17G1 subtype) | | TCTGTTGAGGGTTCATCATCATCATCATCATCATCATCATCATCATCGTCATCATCATCATCATCAACATCGACTGTAGCACCAACTCCAAAGAAAGAAAGAACTGGA**GAGGAAGTAGGTAATCCAGGTTCT**GAAGGTCAGGACGGTAAAGGAGACAATGAAGAAACAGAAGACAATCAGACCGAGAGTACTGTTTCTCAAAATACTTCAGCTCAAACTGAAGGCACAACTACCGAAACCACAGAAGCTGCTCCAAAGAAAGAGTGCGGTACTTCATTTGTTATGTGGTTCGGAGAGGGTGTTCCAGTTGCATCTTTGAAGTGTGGCGACTATACTATGG |
| RPA primers | |  |
| 1 | F01332 | CTCATTATCGTATTACTCTCCGTTATAGTCTC |
|  | R29735 | CTGATTGTCTTCTGTTTCTTCATTGTCTCCTTTAC |
| 2 | F01431 | TCATTATCGTATTACTCTCCGTTATAGTCTC |
|  | R29835 | TCTGATTGTCTTCTGTTTCTTCATTGTCTCCTTTA |
| 3 | F00635 | ATTGTCGCTCATTATCGTATTACTCTCCGTTATAG |
|  | R29634 | TGATTGTCTTCTGTTTCTTCATTGTCTCCTTTAC |
| 4 | F01233 | GCTCATTATCGTATTACTCTCCGTTATAGTCTC |
|  | R29533 | GATTGTCTTCTGTTTCTTCATTGTCTCCTTTAC |
| 5 | F01333* | CTCATTATCGTATTACTCTCCGTTATAGTCTCC |
|  | R32535* | AAGTATTTTGAGAAACAGTACTCTCGGTCTGATTG |
| 6 | F01331 | CTCATTATCGTATTACTCTCCGTTATAGTCT |
|  | R29734 | CTGATTGTCTTCTGTTTCTTCATTGTCTCCTTTA |
| 7 | F01432 | TCATTATCGTATTACTCTCCGTTATAGTCTCC |
|  | R29532 | GATTGTCTTCTGTTTCTTCATTGTCTCCTTTA |
| 8 | F01531 | CATTATCGTATTACTCTCCGTTATAGTCTCC |
|  | R29635 | TGATTGTCTTCTGTTTCTTCATTGTCTCCTTTACC |
| 9 | F01630 | ATTATCGTATTACTCTCCGTTATAGTCTCC |
|  | R29534 | GATTGTCTTCTGTTTCTTCATTGTCTCCTTTACC |
| 10 | F00825 | TGTCGCTCATTATCGTATTACTCTC |
|  | R24025 | ATTACCTACTTCCTCTCCAGTTCTT |
| PCR primers | |  |
| AL3531 | | ATAGTCTCCGCTGTATTC |
| AL3533 | | GAGATATATCTTGGTGCG |
| AL3532 | | TCCGCTGTATTCTCAGCC |
| LX0029 | | CGAACCACATTACAAATGAAGT |
| crRNA | | AAUUUCUACUGUUGUAGAU**agaaccuggauuaccuacuuccuc** |
| FAM-TTATT-Biotin reporter | | 6-FAM-TTATT-Biotin |
| HEX-TTATT-BHQ1 reporter | | HEX-TTATT-BHQ1 |

Underline indicates the PAM region.

Bold characters represent a crRNA binding region and targeted PAM-proximal truncate sequence.

* Corresponds to the optimum RPA primers used in this study.

Table S2. Partial sequences of C. parvum gp60 gene of six subtype families (IIa-IIf) cloned into the pUC57 vectors.

>FJ839880-IIa

ATGAGATTGTCGCTCATTATCGTATTACTCTCCGTTATAGTCTCCGCTGTATTCTCAGCCCCAGCCGTTCCACTCAGAGGAACTTTAAAGGATGTTCCTGTTGAGGGCTCATCATCGTCATCGTCATCGTCATCATCATCATCATCATCATCATCATCATCATCATCATCATCAACATCAACCGTCGCACCAGCAAATAAGGCAAGAACTGGAGAAGACGCAGAAGGCAGTCAAGATTCTAGTGGTACTGAAGCTTCTGGTAGCCAGGGTTCTGAAGAGGAAGGTAGTGAAGACGATGGCCAAACTAGTGCTGCTTCCCAACCCACTACTCCAGCTCAAAGTGAAGGCGCAACTACCGAAACCATAGAAGCTACCTCAAAAGAAGAATGCGGCACTTCATTT

>AF402285-IIb

ATGAGATTGTCGCTCATTATCGTATTACTCTCCGTTATAGTCTCCGCTGTATTCTCAGCCCCAGCCGTTCCGCTGAGAGGCACCTTGAAGGATGTTTCTGTTGAGGGCTCATCATCATCATCATCATCATCATCATCATCATCATCATCAACGACCGTCGCACCAGCTTCAAATAAGGCAAGAACTGGAGAAGACACAGAAGGTAGTCAAGTTTCTGGTGGTACTCCTGAATCTTCTGGCAGCCAGGACACTGAGGAAAGTGAAGACGGTTCCCAAACTAGTACTGTCTCCGAATCCACTACTCCAGCTCAAAGTGAAGGCACAATTACCGAAACCACAGAAGCTGCTCCAAAGAAAGAGTGCGGTACTTCATTT

>FJ839876-IIc

ATGAGATTGTCGCTCATTATCGTATTACTCTCCGTTATAGTCTCCGCTGTATTCTCAGCCCCACCCGTTCCACTCAGAGGCACTTTAAAGGATGTTTCTGTTGAGAGCTCATCGTCATCATCGTCATCGTCAACAACAACCCCCGCACCAGCTCCAAAGAAGGTAAGAGAAAGCGAAGAAGGGAAGAACAGTGAAGATAGTCAAACTCCCGCTAGTCCTGGAAGTGATTCTCAGGATAGCTCTAAAGGAGACGAAGTTGTAGGTGGAGGCGCTTCCGGATCTAGTACCCCAACTCAAGCTGCTGAAAAGGAGCCCGAAACTCCAGAATCTACTCCAAAGGAAGAATGTGGTACTTCATTT

>FJ839877-IId

ATGAGATTGTCGCTCATTATCGTATTACTCTCCGTTATAGTCTCCGCTGTATTCTCAGCCCCAGCCGTTCCACTCAGAGGCACTTTAAAGGACGTTTCTGTTGAGGGTTCATCATCATCATCATCATCATCATCATCATCATCATCATCATCATCATCATCATCGTCATCATCATCATCATCAACATCGACTGTAGCACCAACTCCAAAGAAAGAAAGAACTGGAGAGGAAGTAGGTAATCCAGGTTCTGAAGGTCAGGACGGTAAAGGAGACAATGAAGAAACAGAAGACAATCAGACCGAGAGTACTGTTTCTCAAAATACTTCAGCTCAGACTGAAGGCACAACTACCGAAACCACAGAAGCTGCTCCAAAGAAAGAGTGCGGTACTTCATTT

>GU214369-IIe

ATGAGATTGTCGCTCATTATCGTATTACTCTCCGTTATAGTCTCCGCTGTATTCTCAGCCCCAGCCGTTCCACTCAGAGGCACTTTAAAGGATGCTTCTGTTGAGGGCTCATCATCATCATCATCATCATCGACCACCGTCGCACCAGCTCCAAAGAAAGAAAGAACTGGAGAGGGCGTAGATGGAAAGGACCAAGTAGATAGTACAGGTTCTGATCAGAGCAGTAAAGGAGACACTGAAGGAATCACAGAAGATGGTAAAGAGACCGGAGATACTGTTTCCCAACCCACTACTCCTCCAGATCAAGGTGAGAGCGCAACTCCCGGATCCACGGAAACTACTCCAAAGGAAGAATGCGGTACTTCATTT

>AY738188-IIf

ATGAGATTGTCGCTCATTCCAAGTCGGTATAGTCTCCGCTGTATTCTCAGCCCCGGCCGTTCCACTCAGAGGCACTTTAAAGGATGTTTCTGTTGAGGGCTCATCATCATCATCATCAACAAAAACCATCGCACCAGCAAATAAGGCAAGAACTGTAGAAAACTCAGAAAGTAGTGATAGTCCAGATTCTGGTGCTGGAGGTAGTTCTGGTACTAGCGATTCTAATGCTACCCAGGATTCAACTCAAGGAGGTACTCAAGGAGATAATCAAGAACAAAGTACTGCTAGCCAAGCCACTGCCCCAACTCAAGGTTCTGCTGACACTACTCAGTCCACAGAAACTAATCCGAATGAAGAATGCGGTACTTCATT
